# Manufacturing cycle prediction using structural equation model toward industrial early warning system simulation: The Indonesian case

**DOI:** 10.1016/j.heliyon.2024.e41522

**Published:** 2024-12-28

**Authors:** Tirta Wisnu Permana, Gatot Yudoko, Eko Agus Prasetio

**Affiliations:** School of Business and Management, Institute of Technology Bandung (ITB), Bandung, Indonesia

**Keywords:** Indonesia, Manufacturing cycle, Composite leading indices (CLI), Time series data, PLS-SEM

## Abstract

This study aims to integrate short-term, medium-term, and long-term Composite Leading Indices (CLIs) to establish that interconnected CLIs offer enhanced predictive capabilities compared to individual CLIs. Specifically, it investigates the relationships among CLIs to forecast Indonesia's Manufacturing Cycle (ManC) using Partial Least Squares-Structural Equation Modeling (PLS-SEM).

Building on an extensive literature review, the study employs quarterly data spanning from Q1 2010 to Q2 2022, incorporating five constructs representing key economic sectors influencing the manufacturing cycle. The analysis includes two short-term CLIs: the Short Leading Economic Index (SLEI) and the International Trade Channel (ITC). The SLEI is composed of two indicators, the Manufacturing Purchasing Managers’ Index (PMI) and the Composite Stock Price Index from the Indonesia Stock Exchange, while the ITC comprises nine critical export-import CLIs.

The Fiscal Cycle (FC) is a potential medium-term CLI, including Gross Domestic Product (GDP) per capita, manufacturing investment, oil prices, and the Consumer Price Index (CPI). Meanwhile, the monetary cycle (MC) comprises the Policy Interest and Real Effective Exchange Rates. This research effectively supports the application of PLS-SEM in forecasting the ManC in Indonesia.

## 1 Introduction

A crucial aspect of economic activity, industrialization can drive economic growth in developed nations by creating productive employment opportunities and enhancing living standards. Structural changes shift value-added growth and labor composition from primary to secondary and tertiary sectors. These structural changes increase per capita income and development indicators while promoting job mobility, income growth, and migration from rural to urban areas.

In Indonesia, as in other developing nations, "industry" mostly refers to commercial manufacturing or processing activities that are not related to oil and gas. Indonesian Central Statistics Agency (BPS) 2020 report states that the growth in manufacturing production was slower in 2012 and 2014–2019 than it was in 2013. The processing industry's average output growth from 2015 to 2019 was 4.3 %, which was marginally less than the 5 % national economic growth rate [1]. The manufacturing sector is heavily represented in BPS records even though it only makes up 19.86 % of GDP [2]. However, to support Indonesia's rapid growth, the manufacturing sector must drive the country's structural reform [3].

The necessity of macroeconomic stability was made abundantly clear by the Indonesian economic crisis. The Rupiah's decline in value relative to other currencies served as the first catalyst for the crisis, which resulted in a number of intricate and long-standing underlying issues. Weak corporate management practices, precarious financial conditions, particularly in the banking industry, and investment finance that was excessively dependent on foreign borrowing were all contributing reasons. The COVID-19 pandemic had a long-lasting effect on the Indonesian economy, causing two quarters of economic decline followed by the 2020 recession [4]. In the meanwhile, the industrial sector has declined. The Ministry of Industry claims that the reason for its notable decline is the sharp decline in domestic demand, which normally accounts for around 70 % of the output of the domestic manufacturing sector [5].

The manufacturing sector's decreased relative GDP contribution starting in 2005 is at the root of the current discussion among Indonesian economists over "early deindustrialization." This topic has also been the subject of significant discussion at the Ministry of Industry. Indonesia is now concentrating on improving the balance of payments in specific economic sectors before being resilient to external shocks. Nonetheless, the industrial sector's supply and demand have been adversely impacted by the COVID-19 pandemic. Declining purchasing power limits demand while global supply chain constraints interrupt supply. Because of this, Indonesian industry may need to focus on utilities, production methods, and striking a balance between domestic and international competitiveness in the future. Furthermore, given the complexity of Indonesia's industrial problems, proper planning is necessary to take supply and demand, economic, environmental, and social concerns into account. The nation's economic status is steadily improving, despite the anticipated fall in the manufacturing sector that has given rise to concerns of deindustrialization.

For two reasons, it may be premature for low- and middle-income nations like Indonesia to reduce their manufacturing capacity. First, deindustrialization occurred early in each of their histories, which led to a decline in income levels. Second, economic growth is slowed when manufacturing dynamism is absent [6]. Three common hyphothesis, that is "early deindustrialization," "reprioritization of export guidelines," and "Dutch disease". Those hypotheses are frequently linked to the economies of developing countries, especially those in Latin America. Prior to reaching the same levels of productivity, competitiveness, and per capita income as developed countries, these countries underwent deindustrialization—a conclusion backed by a number of earlier research [[7], [8], [9]].

Despite the significance of these concepts, fiscal policy's impact on an economy's industrial structure has received insufficient attention in the research, both before and after the financial crisis. In response, empirical and theoretical contributions have emerged [[10], [11], [12]].

On the other hand, global regions and individual countries have seen recurring economic oscillations, some of which have escalated into full-fledged economic crises. This repeating pattern catalyzes the growing interest in developing Early Warning Systems (EWS) to inform policymakers in advance, allowing them to mitigate possible economic damage before it deepens [13].

The leading indicators designed to indicate economic recessions and recoveries constitute a widely employed tool in Early Warning Systems (EWSs) [14]. The concept of leading indicators, initially introduced by Mitchell and Burns in the 1930s [15], highlights their ability to signal economic changes at varying time intervals. The Organization for Economic Cooperation and Development (OECD) classifies these indicators into two categories: short-to-medium-term indicators, which provide signals 2–8 months in advance, and long-term indicators, which lead economic changes by more than eight months [16]. Conversely, Babecký et al. [17] propose a more detailed classification system, dividing economic leading indicators into late warnings (1–3 quarters ahead), early warnings (4–8 quarters ahead), and ultra-warnings (over 9 quarters ahead). They further observe that financial indicators generally precede economic cycles (EC) for longer periods compared to macroeconomic variables [18]. Due to their enhanced forecasting accuracy compared to individual indicators [17,18], researchers typically amalgamate leading indicators into CLIs that align with their primary forecasting horizons, such as short-, medium-, and long-term CLIs. Nevertheless, they often serve as distinct early warning tools.

The studies above explored the interaction between monetary and fiscal policies when analyzing their effects on the industrial structure. Indonesian industrialization increased economic growth by emphasizing the shift from primary to secondary and tertiary sectors. The challenges currently facing the Indonesian manufacturing sector are the financial crisis and the impact of the COVID-19 pandemic. “Early deindustrialization” is occurring, requiring an understanding of the interaction between industrial structure and fiscal and monetary policy. Economic fluctuations also occur globally, so an EWS is needed to mitigate potential financial damage.

As a result, there is significant of interest in investigating and empirically determining the impact of financial cycles, monetary cycles, the transmission of international commerce, and short-leading indicators of changes in manufacturing output. These factors exacerbate the degree of deindustrialization brought on by fiscal stimulus in an open economy that targets inflation, with an emphasis on Indonesia in particular.

The purpose of this study is to investigate the links and effects of key macroeconomic factors in Indonesia's manufacturing sector. Because of the opaque nature of Indonesia's macroeconomic policy, this valuable case study provides crucial lessons. While targeting goals like as inflation control and economic growth, the underlying transmission mechanisms are less clear. This study answers three important concerns about the significance of the industrial sector in Indonesian economic development: (i) What is the relationship between Indonesia's manufacturing sector and monetary cycle? (ii) What is the relationship between the manufacturing sector and the financial cycle in Indonesia? (iii) How do these developments interrelate with the international trade cycle?

This study examines the interconnections among Composite Leading Indices (CLIs) to forecast the Manufacturing Cycle (ManC) in Indonesia during the period from Q1 2010 to Q2 2022, using Partial Least Squares-Structural Equation Modeling (PLS-SEM). PLS-SEM is particularly suitable for this analysis as it allows the construction of CLIs through a measurement model and the evaluation of their relationships within a structural model. The research offers a novel approach by utilizing PLS-SEM to forecast the ManC based on the interconnectedness of CLIs. To the best of the authors’ knowledge, this method has seen limited application in early warning system (EWS) studies, particularly in the context of Indonesia, and no prior research has specifically addressed an EWS for the manufacturing sector. This paper draws inspiration from studies on forecasting economic cycles conducted in Thailand [13], acknowledging that specific inquiries remain unanswered.

The conclusions are confined to a macro-level analysis of Indonesia's economic fundamentals and an aggregation approach to examining the industrial sector. The results are not comparable to those of other ASEAN-member or non-member nations with similar characteristics. This study can be compared to other time series analyses in the literature. It also analyzes various studies that investigate the impact of fiscal, monetary, and international trade cycles on industrial structures.

The research is organized as follows: Section 1 introduces the research subject, background, objectives, and questions. Section 2 provides a comprehensive literature review, comparing the research to previous time series analyses and serving as a reference for empirical findings. Section 3 of the study outlines the data and research methodology. Section 4 presents the empirical findings and discusses the results. Finally, Section 5 presents the study conclusions. The outcomes presented in this research will help policymakers formulate strategies for the manufacturing sector, providing valuable insights into the macroeconomic transmission mechanisms that can contribute to sustainable economic growth in Indonesia. The analysis proposed in this research will contribute to the existing literature.

## 2 Literature review

Several studies examined how the manufacturing sector affected economic growth, but they did not empirically examine how it affected other macroeconomic factors. Consistent with other studies, this study examined if, using some of the chosen macroeconomic variables, Indonesia's manufacturing sector has a causal relationship with economic progress [[20], [21], [22]].

The monetary policy transmission mechanism explains the way monetary policy affects real economic activity, the transmission mechanism is the process through which monetary policy decisions are transmitted into real GDP changes and inflation [24]. Clunies-Ross [21] and Jhingan [25] argued that rising per capita incomes lead to economic development. GDP, sometimes used as a proxy for economic growth, represents the economy's ability to produce products and services, eventually contributing to citizens' welfare [26]. Stable growth is critical for increasing GDP, and when economic progress exceeds population expansion, human well-being improves. Furthermore, it promotes per capita output and total productivity, particularly of labor, which enhances the economy's productive potential and wealth development. According to Balami [27], quantitative economic growth metrics include nominal and real output growth, as well as per capita value.

The term "industrialization" describes the labor patterns and output of a nation's manufacturing or secondary industries [28]. Income thresholds, which represent the transition from agrarian to industrial society, might also serve as indicators of this change. Preliminary research indicates that industrialization encouraged sociological rationalization, whereas modernization, extensive energy development, and metallurgical production changed the industry and contributed to economic growth. A variety of strategies, including import substitution, export promotion, and balanced and unbalanced growth, have been utilized to promote industrial development.

Subsector links are essential for economic growth in any country, and are especially important in developing countries [29]. In India, industrialized states are experiencing faster growth via regional hypothetical growth engines. Isiksal and Chimezie's [30] cointegration tests revealed a long-term association between three variables used to assess Nigerian industrialization between 1997 and 2012, with the agriculture industry showing a positive correlation with GDP. These research demonstrated that manufacturing was essential in driving post-1990s growth despite a decreased total percentage of the national economy during the same period.

Several studies have explored the role of manufacturing in contributing to per capita GDP growth, considering factors such as education provision and economic development [[31], [32], [33]]. Furthermore, manufacturing is a driving force of growth in low- and middle-income nations with plentiful human capital, whereas the services sector lacks a comparable growth engine. A five-year GDP regression analysis found a positive association between GDP growth rates and the fraction of the economy represented by industry [34]. This observation supported the argument that modern industry promotes growth, and structural transformation is necessary to accelerate this process in underdeveloped countries. McCausland and Theodossiou [35] studied the impact of manufacturing on productivity and economic growth between 1992 and 2007 in 11 countries: the United Kingdom, the United States, Canada, Australia, Germany, France, Sweden, Greece, Japan, Korea, and Taiwan, using practical empirical estimation methods that include Fixed Effects and Generalized Least Squares. Empirical study showed that industrial output continues influence productivity and economic growth. Kathuria and Natarajan analyzed how manufacturing supported economic growth in 15 Indian states from 1994 to 1995 to 2005–2006, outlining that factor accumulation drives production [33].

Several research papers have examined the impact of manufacturing on economic growth in 88 countries from 1950 to 2005 [32]. Empirical analysis indicated that manufacturing drives economic growth [36]. Furthermore, the interaction rate between manufacturing and education, as well as the income disparity within the manufacturing sector, indicate that the former has a beneficial impact on economic growth. In contrast, the interaction rate between manufacturing and GDP per capita has a largely negative impact. They concluded that manufacturing promotes economic development in developing countries with highly educated workforces.

Marconi et al. [37] analyzed 63 countries statistics to investigate the relationship between increased manufacturing production, economic development, and productivity in high- and middle-income countries between 1990 and 2011. The authors discovered that greater manufacturing production favorably effects economic growth and the development of middle-income countries. Cantore et al. [38] used the Generalized Method of Moments to test Kaldor's law [39] When evaluating the extent to which manufacturing contributed economic growth in 80 nations between 1980 and 2010. The findings indicated that manufacturing growth components are suitable for economic development given that they are divided into structural transformation and employment levels [38]. Structural transformation enhances manufacturing added value due to productivity, while employment increases in response to overall opportunities [38]. Empirical analysis revealed that the manufacturing sector supports economic growth and represents a structural transformation that promotes such expansion [39]. Bolaky [40] identified empirical and theoretical evidence for industrialization as a process that marks the transition from an agricultural to a manufacturing-dominated economy. Industrialization has two dimensions: economic activity and economic production by employing mechanization, methodical techniques, and inventive practices.

Several existing ideas on deindustrialization present opposing views on whether the process is potentially positive or negative [41]. As an illustration, in developed economies, deindustrialization is regarded as economic development. In less developed countries, it denotes economic deterioration, which leads to decreased consumption, production, and failure. This negative deindustrialization can lead to an economic slowdown by lowering income and consumption. Rowthorn and Coutts [9] viewed Marx's thesis of industrial profit decrease as the foundation for deindustrialization. According to this notion, technological advancements might boost efficiency and output. Such improvements may reduce employment by replacing workers with machines and increasing expenditure on capital.

In contrast, a decrease in surplus related to labor expansion will result in a decline in the industry's long-term profitability. To avoid negative deindustrialization, an industry must encourage technical innovation and the development of a highly qualified workforce. A well-developed manufacturing sector demonstrates high productivity [42]. When other factors remain constant, increased manufacturing sector productivity may lower relative production costs, resulting in cheaper pricing. It might cause the proportion of added value within the manufacturing sector to decrease, assuming changing demand for manufactured goods and services. Outsourcing or contraction reduces industrial sector activities while increasing value without weakening economic conditions. Manufacturing benefits from deindustrialization because it is more productive.

Bazen and Thirlwall, as mentioned in Jalilian and Weiss [43], emphasized the importance of focusing on manufacturing sector employees when investigating the relationship between industrialization, job growth, and improved salaries at various worker productivity levels. According to these experts, good deindustrialization does not worsen job losses, whereas negative deindustrialization can lead to increased unemployment. Empirical research demonstrates that inflation contributes to deindustrialization [44] by raising investment costs and lowering commercial earnings, resulting in a reduction in industrial activity. Furthermore, government laws have an impact on deindustrialization since advances in transportation, communications, and information technology encourage manufacturing enterprises to relocate to lower-cost locations, leaving downtown areas for service and finance [31,43].

Dasgupta and Singh [45] identified that in an open economy, industrialization and deindustrialization processes require both domestic and foreign viewpoints. In developing countries, agriculture contributes more to the balance of payments than manufacturing during the early phases of economic expansion. However, as per capita income rises and these countries approach middle-income status, industry gains prominence due to the high demand for produced goods. When domestic manufacturing cannot match the demand, many countries resort to importing these items, potentially negatively affecting their trade balance. Rather than manufacturing, developed economies primary export sectors frequently include knowledge-based services. In the long run, knowledge-intensive services such as information technology, banking, and consulting outperform traditional manufacturing in terms of GDP and job creation. This distinct pattern of economic activity distinguishes developed nations from developing ones, requiring careful consideration when studying economic development and trade dynamics.

Kaldor [39] argues that manufacturing is the fundamental driver of rapid economic growth. A group of 12 industrialized countries was recognized, ranging from the United Kingdom to Japan, as well as numerous developing countries that have achieved sustained and significant growth. The investigation indicated that successful structural adjustments implemented by these high-income countries consistently resulted in deindustrialization, which boosted growth. Several studies have consistently shown that manufacturing considerably boosts economic growth. Nonetheless, there is a scarcity of empirical study on how manufacturing affects other key macroeconomic indicators. Recent analyses have sought to fill this vacuum by focusing on 158 nations from 1950 to 2013, investigating the manufacturing sector's effect on service sector growth rates, savings, and total factor productivity in middle-income countries [39]. Rigorous empirical study was conducted, using Granger panel and panel regression model causalities, and the findings demonstrated that manufacturing did, in fact, encourage service sector growth, improve saving rates, and increase total factor productivity [22]. These findings highlight the manufacturing sector's multifaceted and critical contribution to middle-income country's overall economic development.

A different area of study focuses on the effects of monetary policy on production structures in open economies with flexible exchange rates, highlighting the importance of markup dynamics and corporate behavior in generating economic cycles [46]. Bergin and Corsetti [47] investigated how monetary stability affects a country's comparative advantage using a unique Keynesian two-country model with monopolistically and perfectly competitive traded goods sectors. Their findings underlined the interaction of fiscal and monetary policy in shaping resource allocation and industrial architecture. They found that stabilizing aggregate demand and markups during economic cycle shocks improves a country's comparative advantage, but instability hinders firms from adapting to production due to entry costs and nominal disturbances.

Røisland and Torvik [48] investigated fiscal policy in an open economy model with traded and nontraded goods sectors. Monetary policy targets inflation. They discovered that expansionary monetary policy raises output in both sectors by lowering interest and exchange rates, whereas fiscal expansion increases production in the nontraded sector while decreasing output in traded goods due to currency appreciation. This theoretical framework highlighted the relative benefits of monetary and fiscal policy in stabilizing different sectors of the economy, with monetary policy better suited to stabilizing aggregate output and fiscal policy more effective in putting decentralized production on solid ground. Torvik [49] extended this study to a three-sector model of an oil-exporting economy, demonstrating that fiscal policy's sectoral effects are stronger during unfavorable oil price shocks. The findings indicate that adequate monetary policy responses can offset these negative effects and improve economic sustainability.

Further studies examined fiscal multipliers using various empirical approaches, as summarized by Ramey [50], including structural VARs and narrative methods. Research by Ramey and Zubairy [50], Ben Zeev and Pappa [51], Corsetti et al. [52], and others [53,54] used these methods to analyze the relationship between fiscal policies and economic outcomes. Model-based studies, such as those by Blanchard [55] and Coenen et al. [56], employed DSGE frameworks to assess fiscal stimulus effects in the US and Europe. They discovered that spending multipliers in the first year ranged from 0.7 to 1, but labor tax multipliers were between 0.2 and 0.4. Interestingly, these authors pointed out that expenditure multipliers frequently fall somewhat below 1.0 due to crowding effects from household consumption and net exports. Zubairy [57] applied a medium-scale DSGE model to investigate the US economy and discovered that first-year expenditure multipliers were about one, whereas labor tax multipliers were about 0.3. Cogan et al. [58] updated Smets and Wouters' [59] DSGE model to use data from the United States. Their analysis found that first-year fiscal spending multipliers were typically between 0.6 and 0.7. These model-based studies provide important insights into how fiscal policy affects the economies of the United States and Europe. They offer policymakers and economists with critical information by defining the amount and behavior of fiscal multipliers under various policy tools and economic scenarios.

Specific research into monetary policy's influence on production structures in flexible exchange rate economies underscores the significance of stable demand and markups for resource allocation and trade adjustments. Bergin and Corsetti [47] investigated the consequences of monetary stabilization on a nation's comparative advantage, applying a new Keynesian two-country model. The two trade sectors in this model were optimally and monopolistically competitive, producing differentiated goods in each country. They concluded that efficient monetary policies that can stabilize aggregate demand and markup following business cycle shocks can reinforce a nation's comparative advantage in producing different goods and inducing resource allocation changes between sectors [60]. However, when demand and markup are unstable, companies resist producing alternative goods due to uncertainties linked to entry costs and nominal disturbances. Cavallari [61] developed a two-country DSGE model to analyze the impact of monetary easing, showing that it reduces entry costs, increases the number of operating firms, and expands output on the extensive trade margin. In this regard, Cavallari and d'Addona [62] used a panel VAR model to study the effects of monetary shocks on trade margins across advanced economies, demonstrating that flexible exchange rates smooth fluctuations and reallocate production to previously untapped sectors. These insights illustrate the dynamic interplay between policy interventions and economic structures, informing strategies for stabilization and growth.

## 3 Methodology

**Fig. 1:**
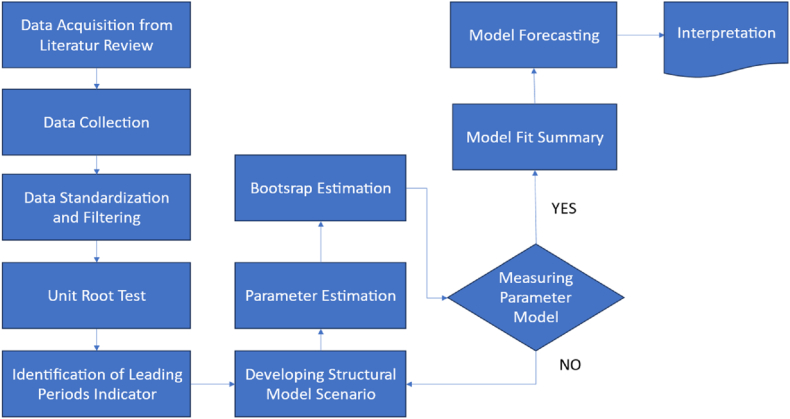
Methodology flowchart.

This paper builds upon the preceding study [13] conducted in Thailand to integrate short-time, medium-time, and long-time Composite Leading Indices (CLIs), aiming to show that interconnected CLIs provide superior forecasting performance compared to individual CLIs. As such, this research explores the relationships among CLIs to predict the Manufacturing Cycle (ManC) in Indonesia through Partial Least Squares-Structural Equation Modeling (PLS-SEM). The PLS-SEM approach follows a general methodological flowchart [75,76], as depicted in Fig. 1.

### 3.1 PLS-SEM

SEM is divided into covariance-based structural equation modeling (CB-SEM) and variance-based (PLS-SEM). There are special differences between these approaches. CB-SEM primarily aims at theoretical confirmation and seeks to estimate model parameters by minimizing the difference between the estimated covariance matrix and the sample data. Therefore, the data used in the model must conform to a normal distribution and come from a large sample. Conversely, PLS-SEM aims to predict the main target construct and maximize the variance explained by unobserved endogenous variables. This method can estimate complex models with arbitrary data distributions and smaller sample sizes.

This study delineates reference series and critical indicators for the ManC based on an extensive literature review. However, many datasets exhibit unit variations, necessitating the standardization of all data to mitigate potential unit effects on the analysis. The outlined procedures adhere to the methodologies prescribed by the OECD CLIs [16].

PLS-SEM is employed to scrutinize the relationships among various sectors, specifically monetary, financial, real, and global, considered CLIs for the ManC to achieve the study's objectives. SEM is particularly suitable for this research because it can assess intricate connections among CLIs.

SEM is highly appropriate for this study as it facilitates the analysis of complex relationships between Composite Leading Indices (CLIs) as unobserved variables and their associated indicators.

Consequently, a considerable body of literature utilizes PLS-SEM for forecasting purposes using small sample sizes [73,74]. Owing to its exceptional strengths, PLS-SEM turned out to be a widely applied statistical technique in numerous academic disciplines [75,76].

### 3.2 Model design

Drawing from the literature review, the Short Leading Economic Index (SLEI), Fiscal Cycle (FC), Monetary Cycle (MC), and International Trade Transmission (ITT) are recognized as potential components of the CLIs for the manufacturing cycle.

The MC, FC, ITT, SLEI, and ManC are latent or unobservable variables, making monitoring them directly challenging. This research identified and employed suitable indicators as proxies for these variables by selecting observable measures representing the latent variables and effectively analyzing relationships and interactions more practically. These selected indicators provide valuable insights into the dynamics and interplay of the economy's monetary, financial, international trade transmission, and manufacturing cycles.

The ManC is characterized by oscillations in activities associated with the industrial sector, reflecting the periodic ups and downs in producing goods and services within this domain. Generally, metrics such as GDP or the Industrial Production Index (IIP) assess this cycle or serve for economic activities within the manufacturing sector [67]. By employing these indicators, analysts gain valuable insights into the overall manufacturing sector's financial performance and dynamics, fostering an understanding of variations in production levels and their broader economic implications.

While no consensus exists regarding defining the financial cycle, it usually involves financial instability. Grinderslev et al. [68] and Wongwachara et al. [69] made specific predictions regarding financial crises. The economic cycle primarily revolves around credit and property prices, although other less significant components may also be considered [64,[66], [67], [68]].

These elements signify the interplay between financing restraint, particularly in the context of property prices [71]. Drehmann et al. [72] developed a financial cycle incorporating credit and asset prices.

Nevertheless, the authors proposed a hypothesis for the financial or fiscal cycles within the Indonesian manufacturing context in this research. This proposed model has three components: GDP per capita, investment, and the consumer price index. By incorporating these indicators, the study aims to comprehensively analyze the financial dynamics within the Indonesian manufacturing sector.

The Bank of Canada initially developed the MC as the national operational target by combining interest and exchange rates [73]. Over time, it garnered widespread usage with numerous organizations, including public and private sector entities, to assess monetary policy stances [74]. In a notable extension, Memon and Jabeen [75] applied the MC concept to the Gulf States to construct a regional MC. They interpreted changes in the MC index as indicative of either the tightening (increasing MC) or easing (decreasing MC) of monetary conditions. Furthermore, they concluded that the MC could predict the GDP, underscoring its relevance and usefulness within economic analysis.

The choice of the United States, along with three major Asian countries—China, Singapore, and Japan, as proxies for ITT is grounded in their economic significance as primary export markets for Indonesia. These economies were selected as ITT representations because demand in these markets typically precedes export activities.

MC's impact was measured by the monetary cycle (interest and exchange rates) on the FC (GDP per capita, Investment, CPI). Subsequently, the FC influenced the ManC, including GDP manufacturing. Buckle et al. [72] stated that the MC displays counter-cyclical behavior within the ManC context. At the same time, the global economy influences the domestic economy through international trade encompassing variables such as imports, exports, importation of capital and consumer goods, raw materials, imports from China, and exports to the USA, Singapore, and Japan. Previous studies referred to variables that establish the relationship between the manufacturing sector and macroeconomic factors, including those conducted by Rowthorn and Coutts [9]. These constitute the leading indicators of the manufacturing sector with selected macroeconomic variables, namely, GDP per capita, Investment (I), Exports, Imports, Imports of capital goods (M_Cap), Imports of raw materials (M_raw), Imports of consumer goods (M_con), Exports to the United States (X_USA), Exports to Japan (X_Japan), Exports to Singapore (X_Sing), Imports from China (M_China), Inflation (CPI), interest rates, and exchange rates. These indicators form the basis for analyzing the dynamics of the manufacturing sector in response to various macroeconomic variables.

**Fig. 2:**
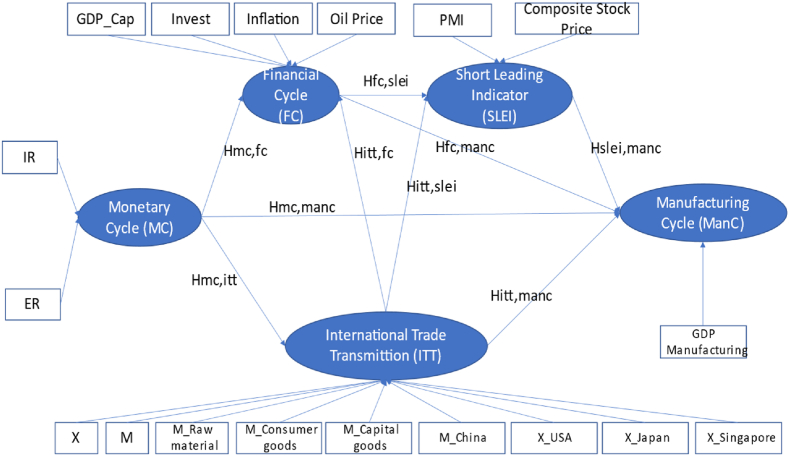
Model framework.

Therefore, this research proposes a model framework elucidating the interrelationships between these CLIs and their elements to predict the ManC with adjustments to apply the hypothesis to the Indonesian case, as depicted in Fig. 2.

• Hypothesis 1: The relationship between the monetary and manufacturing cycles exhibits counter-cyclical behavior with tightening monetary policy, which could directly and indirectly affect the manufacturing cycle. In other words, when monetary policy becomes more restrictive, it will likely dampen manufacturing activity.
• Hypothesis 2: The fiscal cycle exhibits counter-cyclical behavior to the manufacturing cycle. Increased financial instability could directly and indirectly harm manufacturing. Consequently, periods of economic turmoil will probably lead to a downturn in the manufacturing sector.
• Hypothesis 3: The ITT displays procyclical behavior to the manufacturing cycle, with fluctuations in export partner economies that could significantly influence the manufacturing cycle. Therefore, changes in export demand from partner countries should affect manufacturing activity positively.
• Hypothesis 4: The SLEI is a short-term CLI exhibiting procyclical behavior concerning the ManC. Changes in the SLEI have a significant positive effect observable in the subsequent 1–3 quarters.

This study initially employs GDP in manufacturing as a proxy for aggregate manufacturing sector activity to identify potential leading indicators of the ManC from Q1/2010 to Q2/2022. Despite the relatively short duration of the time series data, it satisfies the sample size requirements. PLS-SEM requires at least ten times the number of indicators used to construct the latent variables in the formative measurement model or ten times the highest number of paths leading to latent variables in the structural model [64].

### 3.3 Data

This study draws on CEIC, BPS, Bank Indonesia, Ministry of Industry, and Indonesia Stock Exchange secondary data and analyzes Q1/2010 to Q2/2022 time series data. Real or base year (2010) quarterly data covering most needs was collected and reviewed. Based on background, problems, and objectives, the study uses several relevant variables and units listed in Table 1.

**Table 1:** Variable, indicator, dimension, and sources.

Unobserved variables	Indicators	Definitions	Unit	Sources
Manufacturing Cycle (ManC)	GDP Manufacture (ManC)	Value added of the manufacturing sector	Billions of IDR	BPS, Proceed
Fiscal Cycle (FC)	GDPCap (FC1)	Overall economic growth approximated to real GDP per Capita	Millions of IDR	BPS and CEIC Proceed
	Investment (FC2)	investment approximated to the gross fixed manufacturing investment	Millions of USD	BPS & Ministry of Industry, Proceed
	Inflation (FC3)	Measured using by Bank of Indonesia inflation targeted/consumer price index (CPI)	%	Bank of Indonesia
	Oil Prices (FC4)	The spot price of a barrel of benchmark crude oil	USD	BPS
Monetary Cycle (MC)	Interest rate (MC1)	Bank Indonesia determines the policy for determining the benchmark interest rate	%	Bank of Indonesia
	Exchange rate (MC2)	IDR exchange rate against US Dollar	IDR	Bank of Indonesia
International Trade Transmission (ITT)	Export (ITT1)	Export approximated to the total volume of exports	Millions of USD	BPS & Ministry of Industry, Proceed
	Import (ITT2)	Import approximated to the total volume of imports	Millions of USD	BPS & Ministry of Industry, Proceed
	MCap (ITT3)	Capital goods imports approximated to capital goods imports	Millions of USD	BPS & Ministry of Industry, Proceed
	Mraw (ITT4)	Raw materials imports approximated to raw material imports	Millions of USD	BPS & Ministry of Industry, Proceed
	MCons (ITT5)	Imports of consumer goods approximated to consumer goods	Millions of USD	BPS & Ministry of Industry, Proceed
	X_USA (ITT6)	Exports to the United States approximated to exports to that country	Millions of USD	BPS & Ministry of Industry, Proceed
	X_Japan (ITT7)	Exports to Japan approximated to exports to that country	Millions of USD	BPS & Ministry of Industry, Proceed
	X_Sing (ITT8)	Exports to Singapore approximated to exports to that country	Millions of USD	BPS & Ministry of Industry, Proceed
	M_China (ITT9)	Imports from China approximated to imports from China	Millions of USD	BPS & Ministry of Industry, Proceed
SLEI	PMI (SLEI1)	Manufacturing Purchasing Index	Index	Ministry of Industry & Markit
	IHSG (SLEI2)	Composite Stock Price Index on the Indonesia Stock Exchange	Index	Indonesia Stock Exchange

### 3.4 Data standardization and filtering

This step evaluates each indicator's cyclical pattern. This research follows the concept of growth cycles, defined as economic activity movements [75,76] This study aligns with the procedures outlined by the OECD for constructing CLIs [16]. The results of data transformation are presented in Appendix 1 and Appendix 2.

## 4 Result

This section will investigate the relationship between CLIs and their indicators in forecasting the Manufacturing Cycle (ManC).

### 4.1 Unit root test

The results of the Unit Root Augmented Dickey-Fuller and Phillip Perron test, as depicted in Table 2. Demonstrating that not all indicators are stationary at level. Thus, there is statistical evidence to conclude that some indicators become stationary in their first differences. This further implies that the indicators are integrated into order one. Consequently, were used for further analysis.

**Table 2:** Unit root test transformation and seasonal adjustment data.

Indicators	Order	Correlogram	ADF-test	Philips Pherron - test
INF	I(1)	Stationary	0.0000∗∗∗	0.0000∗∗∗
Exchange rate	I(1)	Stationary	0.0001∗∗∗	0.0001∗∗∗
GDP per Capita	I(1)	Stationary	0.0000∗∗∗	0.0000∗∗∗
Investment	I(1)	Stationary	0.0000∗∗∗	0.0000∗∗∗
Interest rate	I(1)	Stationary	0.0000∗∗∗	0.0000∗∗∗
Import	I(1)	Stationary	0.0000∗∗∗	0.0000∗∗∗
Import of Capital goods	I(1)	Stationary	0.1144	0.0000∗∗∗
Import of Raw material	I(1)	Stationary	0.0000∗∗∗	0.0000∗∗∗
Import from China	I(1)	Stationary	0.0000∗∗∗	0.0000∗∗∗
Import of Consumer goods	I(1)	Stationary	0.0000∗∗∗	0.0000∗∗∗
GDP Manufacturing	I(1)	Stationary	0.0000∗∗∗	0.0000∗∗∗
Export	I(1)	Stationary	0.0000∗∗∗	0.0000∗∗∗
Export to Japan	I(0)	Stationary	0.0045∗	0.0000∗∗∗
Export to Sing	I(1)	Stationary	0.0000∗∗∗	0.0073∗
Export to USA	I(1)	Stationary	0.0000∗∗∗	0.0000∗∗∗
PMI	I(1)	Stationary	0.0000∗∗∗	0.0000∗∗∗
IHSG	I(1)	Stationary	0.0000∗∗∗	0.0001∗∗∗
Oil Price	I(1)	Stationary	0.0000∗∗∗	0.0000∗∗∗

### 4.2 Identify the leading periods of CLIs using PLS-SEM

**Fig. 3:**
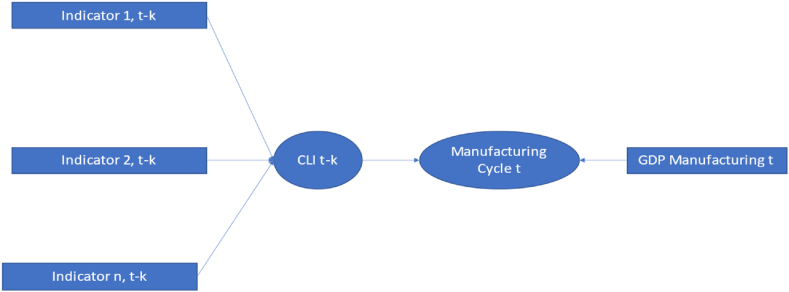
The leading indicator of each CLI.

To roughly identify the leading periods of these CLIs, this study constructs each CLI indicator for the k leading periods (t-k, where k = 0,1, …,12) of ManCt, as shown in Fig. 3. It selects only those values of k that result in an absolute correlation (|R|) between CLIt−k and ManC greater than 0.7, or an R^2^ value of 0.50 or higher.

The results, presented in Table 3, show that SLEIt−k leads ManCt by two periods (k = 2), FCt−k tends to lead ManCt by nine periods (k = 9), and MCt−k can lead ManCt by either nine or twelve periods (k = 9, 12). Additionally, ITTt−k leads ManCt in three or six consecutive periods (k = 3, 6).

**Table 3:** The outcomes validate the leading efficacy of each CLI within the ManC.

*CLI t*−*k*	k leading period producing R^2^ ≥ 0.5
*SLEI t*−k	2
*FC t*−*k*	9
*MC t*−*k*	9,12
*ITT t*−*k*	3, 6

### 4.3 Building an IEWS

**Fig. 4:**
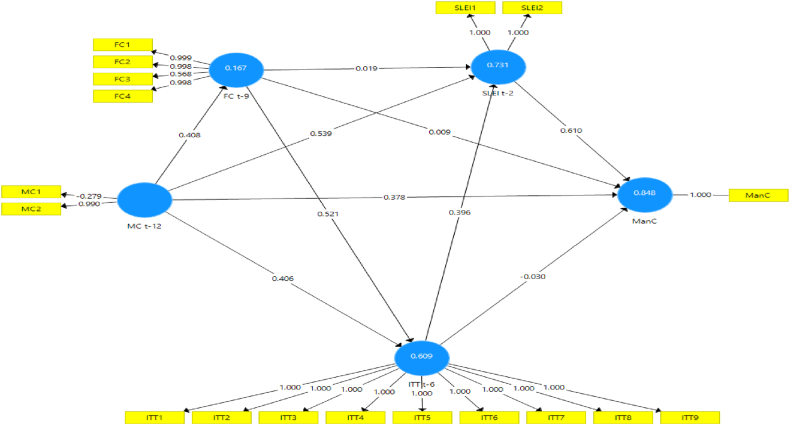
Model-1.

**Fig. 5:**
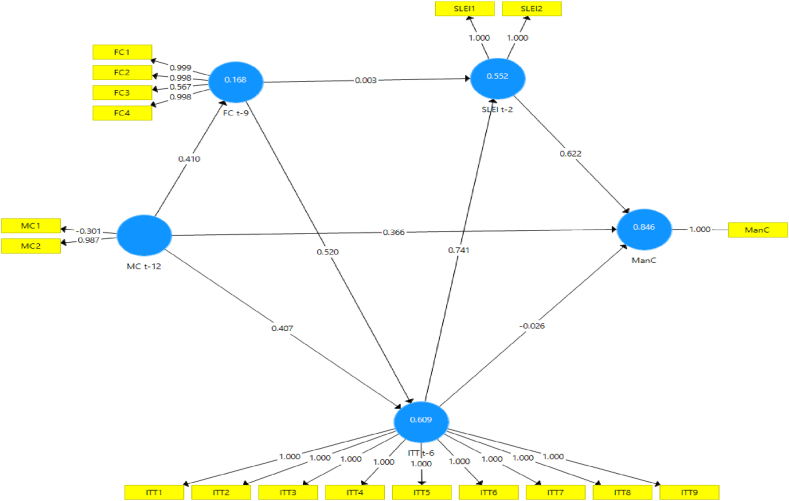
Model-2.

**Fig. 6:**
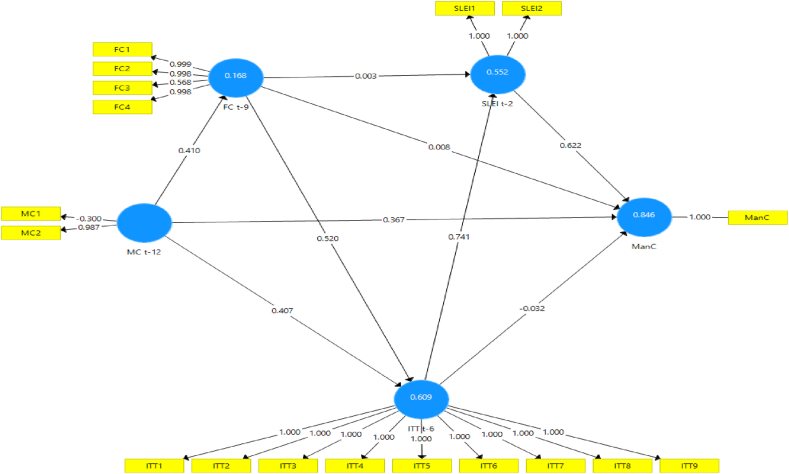
Model-3.

**Fig. 7:**
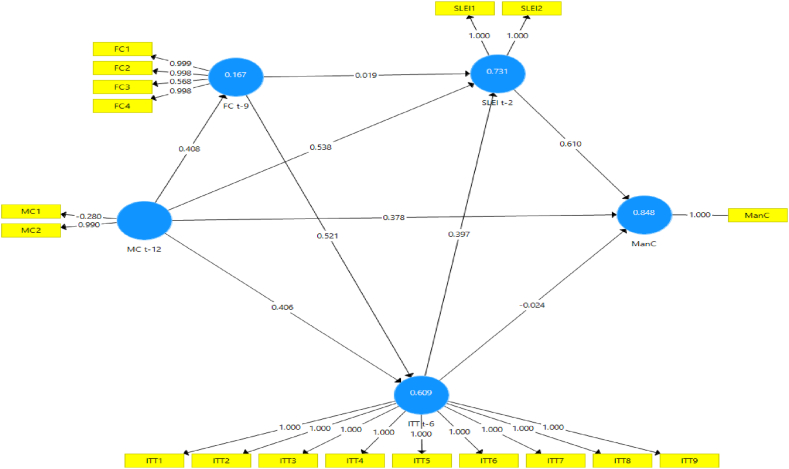
Model-4.

**Fig. 8:**
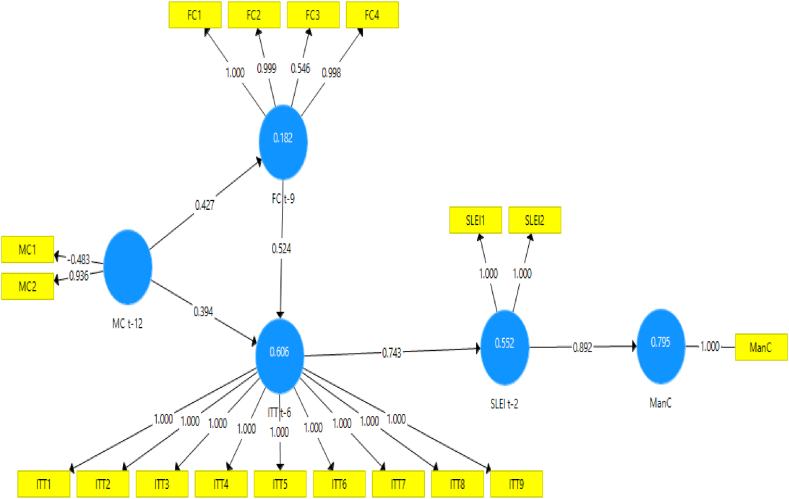
Model-5.

**Fig. 9:**
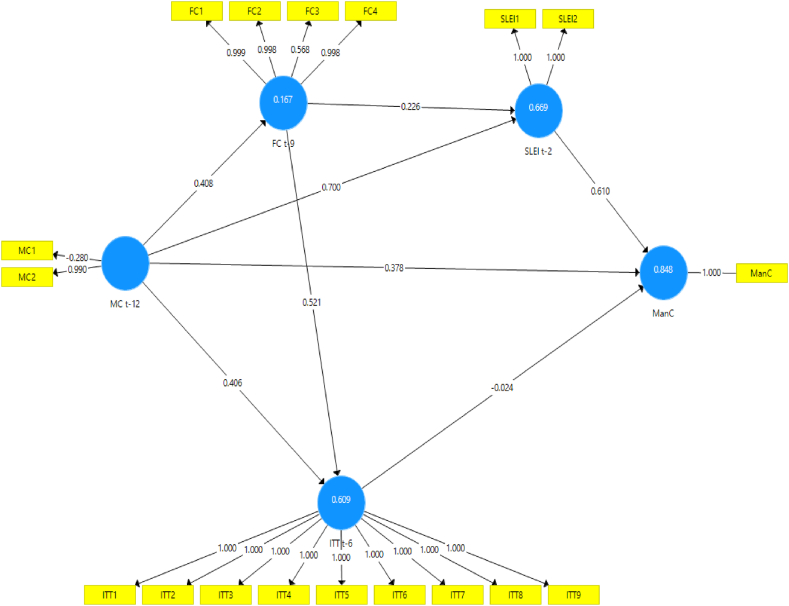
Model-6.

Numerous model scenarios are formulated between CLI t−k at the selected k values to estimate the ManCt. These models are designed to produce the best representation of the ManC construct at time t, as shown in Fig. 4, Fig. 5, Fig. 6, Fig. 7, Fig. 8, Fig. 9. These scenarios probably involve different combinations of CLIs and may incorporate various factors and relationships to enhance the accuracy of the ManC forecasts.

This research eliminates these paths to develop an Industrial Early Warning System (IEWS) with a parsimonious model and chooses two scenario structural models that produce the most optimal parameters such as outer loadings, Variance Inflation Factor (VIF), construct reliability, validity, adjusted R^2^, R^2^, and model fit criteria after considering the lag length. Each scenario's specific details and results must be reviewed to understand the model formulations and their outcomes comprehensively.

### 4.4 Assessing IEWS the result of PLS-SEM

This research examines formative measurements and structural models to evaluate the Industrial Early Warning System (IEWS) using PLS-SEM output for each model scenario and compares them to determine the optimal or best model. Given the nonparametric nature of PLS-SEM that does not assume normality of data, parameters such as outlier loadings, Variance Inflation Factor (VIF), construct reliability, and validity are critical to determining the best model for IEWS.

Based on the PLS-SEM results, model-1, model-2, model-3, model-4, and model-6 have almost the same path and parameter size values, but model-1 has the most optimal R2 and adjusted R2 values. Even though it does not have statistical significance, the decision to maintain these pathways for the sake of practicality and feasibility of implementing EWS while still achieving relatively high explanatory power (as indicated by the R2 value). In contrast to model-5 which has statistical significance, it shows that model simplicity is preferred in this case. Referring to that the author assumes that to maintain all indicators, model-1 and model-5 are considered optimal models in subsequent analysis. A comparison of these measures is presented in Appendix 3.

The next step is to analyze model-1 and model-5 based on the PLS-SEM results presented in Table 4, Table 5, Table 6, Table 7, almost all CLI indicators in model-1 and model-5 have an outer loading value of >0.7, and only FC3 and MC1 have an outer loading value of <0.7. as in Table 4.

**Table 4:** Outer loadings structural Model-1 and Model-5.

	FC t-9		ITT t-6		MC t-12		ManC		SLEI t-2	
	Model-1	Model-5	Model-1	Model-5	Model-1	Model-5	Model-1	Model-5	Model-1	Model-5
FC1	0.999	1								
FC2	0.998	0.999								
FC3	0.568	0.546								
FC4	0.998	0.998								
ITT1			1	1						
ITT2			1	1						
ITT3			1	1						
ITT4			1	1						
ITT5			1	1						
ITT6			1	1						
ITT7			1	1						
ITT8			1	1						
ITT9			1	1						
MC1					−0.28	−0.483				
MC2					0.99	0.936				
ManC							1	1		
SLEI1									1	1
SLEI2									1	1

**Table 5:** Inner VIF values Model-1 and Model-5.

	FC t-9		ITT t-6		MC t-12		ManC		SLEI t-2	
	Model-1	Model-5	Model-1	Model-5	Model-1	Model-5	Model-1	Model-5	Model-1	Model-5
FC t-9			1.2	1.222					1.893	
ITT t-6							2.245		2.555	1
MC t-12	1	1	1.2	1.222			2.695		1.621	
ManC										
SLEI t-2							3.714	1

**Table 6:** Construct reliability and validity Model-1 and Model-5.

	Cronbach's Alpha		rho_A		Composite Reliability		Average Variance Extracted (AVE)	
	Model-1	Model-5	Model-1	Model-5	Model-1	Model-5	Model-1	Model-5
FC t-9	0.931	0.931	1.012	0.998	0.949	0.947	0.828	0.823
ITT t-6	1	1	1	1	1	1	1	1
MC t-12	−0.337	−0.337	0.985	0.38	0.349	0.188	0.53	0.555
ManC	1	1	1	1	1	1	1	1
SLEI t-2	0.999	0.999	0.999	0.999	1	1	0.999	0.999

**Table 7:** R^2^ and R^2^ adjusted Model-1 and Model-5.

	R^2^		R^2^ Adjusted	
	Model-1	Model-5	Model-1	Model-5
FC t-9	0.167	0.182	0.149	0.165
ITT t-6	0.609	0.606	0.592	0.59
ManC	0.848	0.838	0.795	0.791
SLEI t-2	0.731	0.552	0.713	0.543

This study additionally assesses the VIF, a statistical metric, to examine potential multicollinearity issues among the key indicators within the same CLIs. The VIF analysis affirms the absence of multicollinearity concerns, as all values fall below the threshold of 5.00, as delineated in Table 5.

VIF values below 5.00 indicate that the key indicators within the same CLIs are not highly correlated, which is essential for the structural model's reliability.

Based on the results in Table 5, all CLI indicators in model 1 and model 5 have VIF values < 5, proving that all indicators do not have high collinearity, so they will not cause errors in assessing significance.

The evaluation assesses construct reliability and validity by verifying that the Average Variance Extracted (AVE) values align with established criteria. These criteria are the foundation for determining the structural model's reliability and validity, as illustrated in Table 6. AVE is a crucial measure in SEM that assesses the amount of variance captured by the latent construct to the measurement error. AVE values that meet the established criteria demonstrate the reliability and validity of the constructs in the model, further supporting its quality and robustness.

A common measure to establish convergent validity on the construct level is the average variance extracted (AVE). This criterion is the grand mean value of the squared loadings of the indicators associated with the construct (i.e., the sum of the squared loadings divided by the number of indicators). Therefore, the AVE is equivalent to the commonality of a construct. Using the same logic as that used with the individual indicators, an AVE value of 0.50 or higher indicates that, on average, the construct explains more than half of the variance of its indicators. Conversely, an AVE of less than 0.50 indicates that, on average, more error remains in the items than the variance explained by the construct.

Composite reliability is a measure of internal consistency reliability, which, unlike Cronbach's alpha, does not assume equal indicator loadings. It should be above 0.70 (in exploratory research, 0.60 to 0.70 is considered acceptable). Cronbach's alpha measures internal consistency reliability that assumes equal indicator loadings. In the PLS-SEM context, composite reliability is a more suitable criterion. However, Cronbach's alpha still represents a conservative measure of internal consistency reliability.

Based on the results in Table 6, all CLI indicators in model 1 and model 5 have AVE, Cronbach's Alpha, and Composite Reliability values > 0.5, proving that all indicators absorbed more than 50 % of the information from their indicators.

Lastly in the model correlation test, the optimal R-squared (R^2^) value is 0.795, although there are instances where specific paths do not exhibit statistical significance. Nevertheless, this research retains these paths to develop a parsimonious EWS model that yields respective R^2^ and adjusted R-squared (R^2^ adjusted) values of 0.848 and 0.838 for model −1 and 0.795 and 0.791 for model-5, as presented in Table 7. In the next step, models meet the model fit criteria, based on the Standardized Root Mean Square value < 0.10 then all models fit even though based on the Root Mean Square Theta and NFI values, as presented in Appendix 3 are not met so it can be concluded that the model fits the data.

### 4.5 Assessing the hypothesis of IEWS by PLS-SEM

Assessing the formative measurement model entails bootstrap analysis to test the significance of each indicator's weight on its respective constructs. This study validates the hypothesis through bootstrap analysis with 5000 resamples for each construct, as delineated in Table 8, Table 9.

**Table 8:** Bootstrapping Model-1.

	Original Sample (O)	Standard Deviation (STDEV)	Confident Interval 2.5 %	Confident Interval 97.5 %	T Statistics (|O/STDEV|)	P-Values
FC t-9 - > ITT t-6	0.521	0.196	0.179	000	2.656	0.008
FC t-9 - > SLEI t-2	0.019	0.656	−0.273	2.459	0.029	0.976
ITT t-6 - > ManC	−0.024	0.201	−0.097	0.029	0.118	0.906
ITT t-6 - > SLEI t-2	0.397	0.704	−2.126	0.809	0.563	0.573
MC t-12 - > FC t-9	0.408	0.126	0.264	0.924	3.239	0.001
MC t-12 - > ITT t-6	0.406	0.152	0.068	0.638	2.679	0.007
MC t-12 - > ManC	0.378	0.206	0.001	0.761	1.831	0.067
MC t-12 - > SLEI t-2	0.538	0.197	0.157	0.924	2.728	0.006
SLEI t-2 - > ManC	0.610	0.286	0.191	1.000	2.131	0.033

**Table 9:** Bootstrapping Model-5.

	Original Sample (O)	Standard Deviation (STDEV)	Confidence Interval 2.5 %	Confidence Interval 97.5 %	T Statistics (|O/STDEV|)	P-Values
FC t-9 - > ITT t-6	0.524	0.210	0.093	0.953	2.521	0.012
ITT t-6 - > SLEI t-2	0.743	0.114	0.487	0.933	6.156	0.000
MC t-12 - > FC t-9	0.427	0.120	0.335	0.937	3.627	0.000
MC t-12 - > ITT t-6	0.394	0.148	0.080	0.638	2.530	0.012
SLEI t-2 - > ManC	0.892	0.073	0.729	1.000	12.382	0.000

The results indicate no multicollinearity issues among the CLIs and that all path coefficients are statistically significant for model-5, although this is not the case for model-1. This finding further strengthens the reliability and validity of the formative measurement model, confirming that the indicators effectively contribute to their respective constructs without causing multicollinearity problems.

The hypothesis testing results shown in Table 8, Table 9 are as follows.

1. Hypothesis MC → ManC: The MC is a long-term CLI for the ManC regarding structure model-1 and model-5:
  • Model-1: The MC does not directly and statistically significantly impact the ManC, although it directly affects the financial cycle. Therefore, the influence of the MC indirectly affects the ManC through the FC and SLEI. The effect on the manufacturing cycle is 0.408, signifying that an increase in the MC will positively impact the FC during the subsequent three quarters.
  • Model-5: Changes in the MC indirectly impact the ManC through the FC and ITT. The effect on the FC is 0.427, signifying an increase positively in the FC during the subsequent three quarters. Furthermore, the MC's impact on ITT is 0.394, indicating that an increase in the MC will positively impact ITT during the ensuing six quarters.

Therefore, the hypothesis of MC → ManC is supported by an indirect influence, as indicated by Model 5.

2. FC → ManC Hypothesis. The FC is a long-term CLI for the ManC regarding structure model −1 and model −5:
  • Model-1: The FC does not have a statistically significant direct and indirect impact on the ManC but has a direct impact on ITT. The effect on the ITT is 0.521, signifying an increased FC will positively impact ITT during the subsequent three quarters.
  • Model-5: The FC has an indirect effect on the ManC by ITT. The total impact on ITT is 0.524, suggesting that an increase in the FC results in a rise during the subsequent four quarters.

Therefore, the hypothesis of FC → ManC is approved with the indirect influence shown by model-5.

3. Concerning International Trade, this indirectly affects the ManC through SLEI with a total impact of 0.743 on model-5, implying that changes in ITT positively impact ManC during the subsequent two quarters. Therefore, the ITT → ManC hypothesis is supported.
4. Evaluating the SLEI → ManC Hypothesis: The path coefficient for SLEI on ManC is 0.892 on model-5 and 0.601 on model-1, indicating that increased SLEI increases ManC during the subsequent two quarters. Therefore, the SLEI → ManC hypothesis is approved.

### 4.6 IEWS forecasting

This study employs the Root Mean Square Error (RMSE) to compare the forecasts of the ManC. Evidence from Appendix 4 indicates that the IEWS model-5 produces slightly more RMSE than CLIs and structural model-1. However, the IEWS model-1 yields less RMSE than some CLIs in the medium-to long-term, implying that IEWS outperforms the benchmarks. First, IEWS exhibits superior performance over a more extended period than the benchmarks (during the Long-leading period). IEWS generates lower RMSE, indicating better forecasting accuracy than the benchmarks.

This study further assesses the forecasting efficacy of the IEWS using PLS-SEM compared to a linear model. This assessment considers both RMSE and the accuracy of directional predictions. IEWS and the linear model are estimated using subsample data through an increasing window rolling approach. RMSE and correct sign predictions reveal that IEWS model-1 and IEWS model-5 demonstrate superior performance compared to the linear model, both in in-sample and out-of-sample forecasts, as presented in appendices 5 and 6.

## 5 Discussion and conclusion

This research applies PLS-SEM to build an IEWS to estimate the ManC in Indonesia.

### 5.1 Discussion and theoretical contributions

This research introduces an index known as the CLIs of the ManC. Subsequently, the relationships among these CLIs are investigated within the structural framework. The research findings indicate that the IEWS estimated through PLS-SEM involves five constructs: a short-term CLI and SLEI signaling the ManC for the subsequent two quarters. SLEI comprises the PMI and the Indonesian Stock Exchange Composite Index (IHSG), while ITT is constructed from the CLIs of Indonesia's primary export-import partners. Functioning as a medium-term CLI, the FC leads the ManC by nine quarters, incorporating variables such as GDP per Capita, manufacturing investment, oil prices, and the CPI. Meanwhile, the MC serves as a long-term CLI, leading the ManC by twelve quarters.

Regarding theoretical implications, evidence of out-of-sample forecasting accuracy explicitly shows that the IEWS Using PLS-SEM demonstrates superior performance compared to benchmark models (CLIs with uniform weighting and linear modeling) for all short-, medium-, and long-term periods. These findings have significant practical implications, providing an IEWS to estimate the ManC. The insights are relevant to policymakers and businesses when formulating strategic plans. Furthermore, applying PLS-SEM can extend beyond the forecasting of the ManC to predict additional economic indicators. Consequently, this research strongly advocates applying the interrelationships between CLIs through PLS-SEM as an IEWS, confirming that “the interconnection between CLIs is more effective than individual indicators for predicting the ManC.”

### 5.2 Conclusion, limitations, and future directions

Based on a comprehensive literature review, this study uses quarterly data from Q1/2010 to Q2/2022, encompassing five constructs representing economic sectors deemed capable of influencing the CLIs of the ManC. Two short-term CLIs have been incorporated: the SLEI and the ITC. SLEI comprises two indicators, specifically the PMI for Manufacturing and the Composite Stock Price Index on the Indonesia Stock Exchange, while ITC comes from nine pivotal export-import CLIs. The FC is a potential medium-term CLI, incorporating GDP per Capita, manufacturing investment, oil prices, and the CPI. Meanwhile, the MC comprises the Policy Interest and Real Effective Exchange rates.

MC does not have a direct and statistically significant impact on ManC, although MC directly impacts the financial cycle. Therefore, the influence of MC indirectly affects ManC through FC and SLEI.

The total influence of MC on the financial cycle (FC) is 0.408, indicating that an increase in MC will positively affect FC over the next three quarters. Model-5 findings**,** changes in MC indirectly affect ManC through FC and ITT. The total influence of MC on FC is 0.427, suggesting a positive impact of MC on FC over the next three quarters. Additionally, the impact of MC on ITT is 0.394, indicating a positive influence over the next six quarters. FC does not have a statistically significant direct or indirect impact on ManC. However, it directly influences ITT, with a total effect of 0.521, signifying that an increase in FC positively affects ITT over the next three quarters. The total impact of FC on ITT is 0.524, demonstrating that a rise in FC leads to an increase in ITT over the next four quarters.

Regarding international trade, ITT indirectly influences ManC through SLEI, with a total effect of 0.743 in Model-5. This suggests that changes in ITT positively affect ManC over the next two quarters. Moreover, an increase in SLEI results in a rise in ManC over the subsequent two quarters.

This study employs the Root Mean Square Error (RMSE) metric to compare ManC estimation accuracy. Evidence from Appendix 3 reveals that the IEWS model-5 generates slightly higher RMSE compared to the CLI and model-1. However, the IEWS model-1 achieves lower RMSE than certain CLIs in medium- and long-term horizons, demonstrating that the IEWS outperforms the benchmark models.

Firstly, the IEWS exhibits superior performance over extended periods, particularly during the long-leading phase. It achieves a lower RMSE, indicating higher forecast accuracy relative to the benchmark models. The study further evaluates the forecasting capability of IEWS, developed using PLS-SEM, in comparison with linear models. This evaluation incorporates RMSE and directional prediction accuracy as performance metrics.

An increasing rolling window approach is used to estimate IEWS and linear models with subsampled data. These models are subsequently extrapolated to out-of-sample data for validation. The RMSE outcomes and correct sign predictions presented in Appendix 4, Appendix 5 illustrate that IEWS model-1 and IEWS model-5 outperform linear models in both in-sample and out-of-sample forecasts.

These findings offer critical indicators and an Early Warning System (EWS) to assist policymakers and businesses in strategic planning for estimating ManC. Furthermore, PLS-SEM demonstrates its utility in estimating other economic indicators. This research successfully establishes the integration of CLIs through PLS-SEM as an IEWS, affirming its effectiveness in forecasting ManC in Indonesia and supporting the broader application of PLS-SEM in economic forecasting.

This research has several limitations that provide suggestions for future studies. The first is the limited generalizability of our findings. Our sample from the aggregate manufacturing in Indonesia limits the generalizability of our findings to other contexts. Indonesia, chosen as a valuable case study, offers insightful lessons due to the often-opaque nature of its macroeconomic policies. Therefore, we chose manufacturing in Indonesia because it is suitable for testing various changes in the industry and external environments. However, our moderated mediation model's four hypotheses might also apply to other developing countries if macroeconomic factors are crucial to their manufacturing industry's recovery and resilience. Therefore, we suggest future studies explore and test the impact of other macroeconomic variables in different sectors or country contexts, such as large firms, sectoral manufacturing, or SMEs.

Second, although this research provides valuable insights into the causal relationships between macroeconomic factors and the manufacturing sector in Indonesia, further research is needed. Future studies should explore the impact of other macroeconomic factors, investigate sector-specific dynamics, and conduct longitudinal analyses to understand the dynamics and drivers of the manufacturing sector, whether based on large companies, sectoral manufacturing, or SMEs in the industry. Conducting longitudinal analyses can provide better insight into what drives proactive behavior and how the mechanism inspires employees to change in the post-pandemic era when the innovative workplace transforms.

## CRediT authorship contribution statement

**Tirta Wisnu Permana:** Writing – review & editing, Writing – original draft, Visualization, Methodology, Formal analysis, Data curation, Conceptualization. **Gatot Yudoko:** Supervision, Methodology, Conceptualization. **Eko Agus Prasetio:** Supervision, Methodology, Conceptualization.

## Data availability statement

The authors of this study are not authorized to share the data without prior permission. However, the data supporting the findings of this study can be obtained from the corresponding author upon request. We apologize for any inconvenience this may cause and appreciate your understanding.

## Declaration of competing interest

The authors declare the following financial interests/personal relationships which may be considered as potential competing interests:Tirta wisnu wisnu permana reports was provided by Ministry of Industry Republic of Indonesia. Tirta wisnu wisnu permana reports a relationship with Ministry of Industry of the Republic of Indonesia that includes: employment. If there are other authors, they declare that they have no known competing financial interests or personal relationships that could have appeared to influence the work reported in this paper.

## Figures and Tables

**Table:** 

	Ln_Manf GDP	Ln_ GDPCap	Ln_ Man Invs	Ln_CPI	Ln_Oilprices	Ln_Interest rate	ln_Exchange Rate	Ln _Import consumer goods	Ln_Import Raw material goods	Ln_Impor capital goods	Ln_Export	Ln_Import	Ln_Export to USA	Ln_Export to Singapura	Ln_Export to Japan	Ln_Import from China	ln_PMI	Ln_IHSG
Year	ManC	FC1	FC2	FC3	FC4	MC1	MC2	ITT1	ITT2	ITT3	ITT4	ITT5	ITT6	ITT7	ITT8	ITT9	SLEI1	SLEI2
2010q1	33,367071	15,77538	28,005826	3,332413	13,70922	2,618068	9,09142	30,59282	32,89823	31,597756	33,385253	33,21459	30,991307	31,111	31,5627	31,25931841	3,898892043	7,880694739
2010q2	33,385454	15,81523	29,728968	3,392122	13,8126	2,625581	9,09252	30,7398	33,01006	31,618184	33,426277	33,31157	31,038703	31,0809	31,62667	31,46465141	3,921112283	7,970382583
2010q3	33,415428	15,85311	29,463702	3,383159	13,7958	2,624364	9,10961	30,73944	33,03264	31,840973	33,48081	33,37244	31,128292	30,87552	31,68894	31,51558665	3,915464302	8,076355795
2010q4	33,425025	15,83172	29,780153	3,393842	13,8057	2,625081	9,12414	30,88379	33,17961	31,856315	33,694627	33,49218	31,227126	31,15232	31,86549	31,57065191	3,911781713	8,195154015
2011q1	33,451706	15,79326	29,639616	3,389112	13,69898	2,617321	9,08118	30,97643	33,16056	31,734356	33,619681	33,46273	31,244777	31,03613	31,8467	31,48796898	3,898892043	8,1660422
2011q2	33,481018	15,83115	30,005575	3,401383	13,77009	2,622499	9,05001	30,90937	33,30539	31,784862	33,747865	33,57517	31,194522	31,38283	32,01833	31,72344366	3,898892043	8,255408772
2011q3	33,530362	15,86662	29,777976	3,393769	13,73962	2,620284	9,05343	31,0551	33,30839	31,937617	33,758429	33,61511	31,196065	31,56375	31,94476	31,69913546	3,921112283	8,253363313
2011q4	33,547004	15,84456	30,104566	3,404677	13,78283	2,623424	9,10127	31,06551	33,34732	32,133617	33,761657	33,68322	31,200828	31,22189	31,88178	31,76474176	3,915464302	8,236413377
2012q1	33,541808	15,83847	29,725415	3,392002	13,85461	2,628618	9,08825	31,02995	33,31396	32,02731	33,693387	33,63464	31,130225	31,18117	31,93379	31,70928404	3,911781713	8,298079343
2012q2	33,580937	15,87729	30,158778	3,406476	13,80618	2,625116	9,13701	31,07456	33,47337	32,187893	33,740687	33,78624	31,191346	31,35066	31,88786	31,93555694	3,903656845	8,291474076
2012q3	33,611211	15,91023	30,493364	3,417509	13,82012	2,626126	9,15199	31,05065	33,37617	32,087043	33,704556	33,69334	31,193675	31,31251	31,85901	31,83837676	3,934921594	8,332086079
2012q4	33,626266	15,88752	30,097333	3,404437	13,84467	2,6279	9,16911	31,12184	33,49529	32,154203	33,74318	33,79888	31,176971	31,40819	31,82669	31,92400595	3,938807971	8,369707286
2013q1	33,633008	15,87867	30,022206	3,401937	13,86752	2,62955	9,17175	30,93656	33,4534	31,937598	33,710873	33,71603	31,248037	31,41394	31,82826	31,78061368	3,921978571	8,461714167
2013q2	33,674597	15,91793	30,40298	3,414541	13,79436	2,62426	9,18019	31,18431	33,51464	32,001547	33,724527	33,79037	31,257434	31,25996	31,87743	31,97910262	3,940185978	8,511952416
2013q3	33,690242	15,95019	30,062179	3,403268	13,94511	2,635129	9,27381	31,20923	33,54379	32,061293	33,755448	33,82439	31,370745	31,26027	31,80961	32,0579243	3,907591959	8,383405918
2013q4	33,716753	15,92813	30,186908	3,407408	14,03345	2,641444	9,36595	31,29663	33,64863	32,142292	33,972941	33,92391	31,475515	31,63672	32,01337	32,09567455	3,925474172	8,377259941
2014q1	33,737128	15,9152	30,039319	3,402507	14,03129	2,641289	9,37985	31,1902	33,60121	32,078976	33,89408	33,86967	31,486157	31,6725	31,86385	32,07183775	3,92286693	8,434336807
2014q2	33,786648	15,95277	30,121548	3,405241	14,02311	2,640706	9,36099	31,28666	33,66084	32,118137	33,880319	33,92849	31,507532	31,43414	31,88453	32,16752658	3,952585613	8,491029624
2014q3	33,802088	15,98497	30,557331	3,419605	13,98706	2,638133	9,37338	31,2522	33,62282	32,077853	33,878137	33,89036	31,525807	31,52571	31,79662	32,08618861	3,931200132	8,541120894
2014q4	33,828962	15,96401	30,475547	3,416925	13,71283	2,618332	9,4129	31,2986	33,64506	32,109553	33,903714	33,91589	31,575338	31,4953	31,88983	32,23048472	3,876751211	8,547811756
2015q1	33,844704	15,9489	30,490479	3,417414	13,37503	2,59339	9,45755	31,1131	33,50269	32,049367	33,845703	33,78444	31,654183	31,39875	31,8403	32,20083975	3,860118893	8,597751028
2015q2	33,892088	15,9856	30,87228	3,429859	13,57552	2,608268	9,48293	31,26489	33,54461	32,024436	33,879287	33,82299	31,589386	31,38651	31,61779	32,18824589	3,853828134	8,531323684
2015q3	33,911677	16,01818	30,629198	3,421954	13,41458	2,596342	9,53733	31,21945	33,50639	32,009624	33,865558	33,78813	31,641679	31,37439	31,72617	32,18864956	3,864504962	8,414499044
2015q4	33,923687	16,00077	30,888405	3,430381	13,21094	2,581045	9,52942	31,29821	33,49185	32,100888	33,812623	33,79959	31,615839	31,33476	31,72337	32,29891939	3,860716195	8,411436005
2016q1	33,928657	15,98583	30,868125	3,429724	12,91894	2,558694	9,51245	31,384	33,39221	31,904062	33,753529	33,69971	31,561833	31,25852	31,63906	32,20391632	3,899950424	8,464600105
2016q2	33,969261	16,02511	30,859474	3,429444	13,24326	2,583489	9,49681	31,32082	33,4639	31,908638	33,815006	33,74789	31,683584	31,42338	31,56098	32,27954285	3,9344366	8,493726592
2016q3	33,983372	16,05597	30,83819	3,428754	13,24181	2,58338	9,48303	31,2709	33,40299	31,883131	33,759671	33,69367	31,546599	31,22893	31,56486	32,1588558	3,910021003	8,579659567
2016q4	33,986917	16,03766	31,077007	3,436468	13,3526	2,591711	9,49149	31,40649	33,52438	32,053968	33,905605	33,82456	31,657664	31,07078	31,67792	32,38428627	3,894537691	8,573457765
2017q1	34,000792	16,0237	30,934645	3,431877	13,44952	2,598943	9,49916	31,40099	33,54494	31,949881	33,929448	33,82292	31,708659	31,31408	31,64575	32,27419073	3,912896747	8,597168266
2017q2	34,034532	16,06302	30,846191	3,429013	13,3773	2,593559	9,49629	31,48182	33,51163	31,882027	33,889893	33,79512	31,656072	31,30045	31,60868	32,32109308	3,921113821	8,657138363
2017q3	34,063727	16,09438	30,670403	3,423298	13,40936	2,595953	9,49778	31,44765	33,62681	32,112534	33,991033	33,9146	31,752397	31,46731	31,77097	32,42841226	3,910287698	8,677374337
2017q4	34,061707	16,07726	30,895981	3,430626	13,59655	2,609816	9,51306	31,59278	33,73543	32,255396	34,052981	34,03108	31,75007	31,43662	31,83075	32,60799193	3,910928207	8,716785633
2018q1	34,075627	16,06247	30,694595	3,424087	13,68634	2,616398	9,51581	31,61099	33,7349	32,1904	34,028238	34,02232	31,763806	31,43645	31,84839	32,5637653	3,925184595	8,773995143
2018q2	34,103235	16,1037	30,840134	3,428817	13,82	2,626117	9,5435	31,71159	33,78803	32,211107	34,042656	34,0755	31,745005	31,42419	31,83054	32,61582769	3,935961486	8,687072047
2018q3	34,139252	16,13414	30,468503	3,416693	13,89457	2,631498	9,58898	31,83684	33,93058	32,378169	34,164646	34,21876	31,90602	31,35379	31,94715	32,79317578	3,931807013	8,695699479
2018q4	34,135758	16,11709	30,64708	3,422537	13,82371	2,626386	9,60323	31,80899	33,95087	32,40958	34,134288	34,23719	31,83907	31,60417	31,84391	32,88265219	3,925992143	8,704075001
2019q1	34,146472	16,1015	30,409783	3,414764	13,69819	2,617264	9,55669	31,50907	33,714	32,196424	33,999959	33,99881	31,715779	31,43643	31,72098	32,63201763	3,919991175	8,776736661
2019q2	34,164646	16,14263	30,731153	3,425277	13,79025	2,623962	9,57027	31,69058	33,72874	32,153036	33,971945	34,01926	31,721	31,39707	31,62194	32,62761409	3,92920783	8,754796915
2019q3	34,202212	16,17266	30,307263	3,411387	13,69251	2,616849	9,56113	31,72823	33,74589	32,344913	34,059009	34,06748	31,824595	31,61452	31,67057	32,74865281	3,896570901	8,747673867
2019q4	34,189667	16,1551	30,618877	3,421617	13,70014	2,617406	9,55681	31,83942	33,75983	32,281391	34,047181	34,07795	31,82414	31,4228	31,65303	32,79363174	3,880876276	8,729056614
2020q1	34,182855	16,11846	30,616309	3,421533	13,50782	2,603269	9,56835	31,57765	33,68238	32,060106	34,022352	33,9595	31,866524	31,52656	31,60553	32,49842731	3,88841314	8,577452696
2020q2	34,119455	16,07567	30,766839	3,426437	12,90962	2,557973	9,6206	31,61301	33,48129	31,97932	33,888272	33,80126	31,668416	31,12328	31,44852	32,57070096	3,457367653	8,47466189
2020q3	34,178176	16,1249	30,602333	3,421076	13,37128	2,593109	9,60181	31,5567	33,48017	32,099126	34,031335	33,81479	31,91834	31,28844	31,50443	32,63798396	3,877431561	8,534256973
2020q4	34,189442	16,12094	30,651475	3,422681	13,36253	2,592454	9,57896	31,70168	33,59885	32,221259	34,134365	33,93688	31,933792	31,18723	31,4956	32,71578392	3,910021003	8,625731433
2021q1	34,197644	16,10393	30,765256	3,426386	13,66288	2,614683	9,56496	31,70308	33,77658	32,165272	34,179103	34,05828	32,011185	31,2376	31,7024	32,79052629	3,953164949	8,704484047
2021q2	34,215886	16,13646	30,780802	3,426891	13,79144	2,624048	9,58051	31,8468	33,89706	32,194721	34,291465	34,16782	32,083332	31,37276	31,65887	32,90257281	3,997588894	8,695538409
2021q3	34,250382	16,15182	30,444773	3,415914	13,86355	2,629263	9,57885	31,97107	33,88647	32,224816	34,420041	34,17698	32,207933	31,37794	31,86547	32,92884076	3,814042597	8,727307213
2021q4	34,26751	16,16241	31,088099	3,436825	13,93803	2,634621	9,57067	32,0992	34,03788	32,448922	34,503005	34,33652	32,311631	31,45871	31,92129	33,11177503	4,004906	8,790104714
2022q1	34,293568	16,14331	30,872665	3,429871	13,85585	2,628708	9,57116	31,81961	34,07125	32,439053	34,486257	34,33417	32,347134	31,53137	32,01847	33,0609163	3,952524949	8,833985616
2022q2	34,299923	16,17985	31,309223	3,443913	13,95306	2,635699	9,5857	31,90056	34,14669	32,393305	34,626214	34,39276	32,251166	31,79637	32,13805	33,10508503	3,931171824	8,867355387

**Table:** 

Year	Ln_Manf GDP_SA	Ln_GDPCap_SA	Ln_Man Invest_SA	Ln_CPI_SA	Ln_Oilprices_SA	Ln_Interest rate_SA	Ln_Exc_SA	Ln_Impor consumer goods_SA	Ln_Import Raw material goods _SA	Ln_Impor of capital goods _SA	Ln_Export_SA	Ln_Import_SA	Ln_Export to USA _SA	Ln_Export to Singapore_SA	Ln_Export to Japan_SA	Ln_Import from China_SA	Ln_PMI_SA	Ln_IHSG_SA
	ManC_SA	FC1_SA	FC2_SA	FC3_SA	FC4_SA	MC1_SA	MC2_SA	ITT1_SA	ITT2_SA	ITT3_SA	ITT4_SA	ITT5_SA	ITT6_SA	ITT7_SA	ITT8_SA	ITT9_SA	SLEI1_SA	SLEI2_SA
2010q1	33,4	15,8	28	3,3	13,7	2,6	9,1	30,6	32,9	31,6	33,4	33,2	31	31,1	31,6	31,3	3,9	7,9
2010q2	33,4	15,8	29,7	3,4	13,8	2,6	9,1	30,7	33	31,6	33,5	33,3	31	31,1	31,6	31,5	3,9	8
2010q3	33,4	15,8	29,5	3,4	13,8	2,6	9,1	30,7	33	31,8	33,5	33,4	31,1	30,9	31,7	31,5	3,9	8,1
2010q4	33,4	15,8	29,8	3,4	13,8	2,6	9,1	30,9	33,2	31,9	33,7	33,5	31,2	31,2	31,9	31,6	3,9	8,2
2011q1	33,5	15,8	29,6	3,3	13,7	2,6	9,1	31	33,2	31,7	33,6	33,5	31,2	31	31,8	31,5	3,9	8,2
2011q2	33,5	15,8	30	3,4	13,8	2,6	9,1	30,9	33,3	31,8	33,7	33,6	31,2	31,4	32	31,7	3,9	8,3
2011q3	33,5	15,8	29,8	3,4	13,7	2,6	9,2	31,1	33,3	31,9	33,8	33,6	31,2	31,6	31,9	31,7	3,9	8,3
2011q4	33,5	15,9	30,1	3,4	13,8	2,6	9,2	31,1	33,3	32,1	33,8	33,7	31,2	31,2	31,9	31,8	3,9	8,2
2012q1	33,5	15,9	29,7	3,4	13,9	2,6	9,2	31	33,3	32	33,7	33,6	31,1	31,2	31,9	31,7	3,9	8,3
2012q2	33,6	15,9	30,2	3,4	13,8	2,6	9,2	31,1	33,5	32,2	33,7	33,8	31,2	31,4	31,9	31,9	3,9	8,3
2012q3	33,6	15,9	30,5	3,4	13,8	2,6	9,3	31,1	33,4	32,1	33,7	33,7	31,2	31,3	31,9	31,8	3,9	8,3
2012q4	33,6	15,9	30,1	3,4	13,8	2,6	9,4	31,1	33,5	32,2	33,7	33,8	31,2	31,4	31,8	31,9	3,9	8,4
2013q1	33,6	15,9	30	3,4	13,9	2,6	9,4	30,9	33,5	31,9	33,7	33,7	31,2	31,4	31,8	31,8	3,9	8,5
2013q2	33,7	15,9	30,4	3,4	13,8	2,6	9,4	31,2	33,5	32	33,7	33,8	31,3	31,3	31,9	32	3,9	8,5
2013q3	33,7	15,9	30,1	3,4	13,9	2,6	9,4	31,2	33,5	32,1	33,8	33,8	31,4	31,3	31,8	32,1	3,9	8,4
2013q4	33,7	15,9	30,2	3,4	14	2,6	9,4	31,3	33,6	32,1	34	33,9	31,5	31,6	32	32,1	3,9	8,4
2014q1	33,7	15,9	30	3,4	14	2,6	9,5	31,2	33,6	32,1	33,9	33,9	31,5	31,7	31,9	32,1	3,9	8,4
2014q2	33,8	15,9	30,1	3,4	14	2,6	9,5	31,3	33,7	32,1	33,9	33,9	31,5	31,4	31,9	32,2	3,9	8,5
2014q3	33,8	16	30,6	3,4	14	2,6	9,5	31,3	33,6	32,1	33,9	33,9	31,5	31,5	31,8	32,1	3,9	8,5
2014q4	33,8	16	30,5	3,4	13,7	2,6	9,5	31,3	33,6	32,1	33,9	33,9	31,6	31,5	31,9	32,2	3,9	8,5
2015q1	33,8	16	30,5	3,4	13,4	2,6	9,5	31,1	33,5	32	33,8	33,8	31,7	31,4	31,8	32,2	3,9	8,6
2015q2	33,8	16	30,9	3,4	13,6	2,6	9,5	31,3	33,5	32	33,9	33,8	31,6	31,4	31,6	32,2	3,9	8,5
2015q3	33,9	16	30,6	3,4	13,4	2,6	9,5	31,2	33,5	32	33,9	33,8	31,6	31,4	31,7	32,2	3,9	8,4
2015q4	33,9	16	30,9	3,4	13,2	2,6	9,5	31,3	33,5	32,1	33,8	33,8	31,6	31,3	31,7	32,3	3,9	8,4
2016q1	33,9	16	30,9	3,4	12,9	2,6	9,5	31,4	33,4	31,9	33,8	33,7	31,6	31,3	31,6	32,2	3,9	8,5
2016q2	33,9	16	30,9	3,4	13,2	2,6	9,5	31,3	33,5	31,9	33,8	33,7	31,7	31,4	31,6	32,3	3,9	8,5
2016q3	34	16	30,8	3,4	13,2	2,6	9,5	31,3	33,4	31,9	33,8	33,7	31,5	31,2	31,6	32,2	3,9	8,6
2016q4	34	16	31,1	3,4	13,4	2,6	9,5	31,4	33,5	32,1	33,9	33,8	31,7	31,1	31,6	32,4	3,9	8,6
2017q1	34	16	30,9	3,4	13,4	2,6	9,5	31,4	33,5	31,9	33,9	33,8	31,7	31,3	31,6	32,3	3,9	8,6
2017q2	34	16,1	30,8	3,4	13,4	2,6	9,5	31,5	33,5	31,9	33,9	33,8	31,7	31,3	31,6	32,3	3,9	8,7
2017q3	34	16,1	30,7	3,4	13,4	2,6	9,5	31,4	33,6	32,1	34	33,9	31,8	31,5	31,8	32,4	3,9	8,7
2017q4	34,1	16,1	30,9	3,4	13,6	2,6	9,5	31,6	33,7	32,3	34,1	34	31,8	31,4	31,8	32,6	3,9	8,7
2018q1	34,1	16,1	30,7	3,4	13,7	2,6	9,5	31,6	33,7	32,2	34	34	31,8	31,4	31,8	32,6	3,9	8,8
2018q2	34,1	16,1	30,8	3,4	13,8	2,6	9,5	31,7	33,8	32,2	34	34,1	31,7	31,4	31,8	32,6	3,9	8,7
2018q3	34,1	16,1	30,5	3,4	13,9	2,6	9,6	31,8	33,9	32,4	34,2	34,2	31,9	31,4	31,9	32,8	3,9	8,7
2018q4	34,1	16,1	30,6	3,4	13,8	2,6	9,6	31,8	34	32,4	34,1	34,2	31,8	31,6	31,8	32,9	3,9	8,7
2019q1	34,1	16,1	30,4	3,4	13,7	2,6	9,6	31,5	33,7	32,2	34	34	31,7	31,4	31,7	32,6	3,9	8,8
2019q2	34,1	16,1	30,7	3,4	13,8	2,6	9,6	31,7	33,7	32,2	34	34	31,7	31,4	31,6	32,6	3,9	8,8
2019q3	34,2	16,2	30,3	3,4	13,7	2,6	9,6	31,7	33,7	32,3	34,1	34,1	31,8	31,6	31,7	32,7	3,9	8,7
2019q4	34,2	16,1	30,6	3,4	13,7	2,6	9,6	31,8	33,8	32,3	34	34,1	31,8	31,4	31,7	32,8	3,9	8,7
2020q1	34,2	16,1	30,6	3,4	13,5	2,6	9,6	31,6	33,7	32,1	34	34	31,9	31,5	31,6	32,5	3,9	8,6
2020q2	34,1	16,1	30,8	3,4	12,9	2,6	9,6	31,6	33,5	32	33,9	33,8	31,7	31,1	31,4	32,6	3,5	8,5
2020q3	34,2	16,1	30,6	3,4	13,4	2,6	9,6	31,6	33,5	32,1	34	33,8	31,9	31,3	31,5	32,6	3,9	8,5
2020q4	34,2	16,1	30,7	3,4	13,4	2,6	9,6	31,7	33,6	32,2	34,1	33,9	31,9	31,2	31,5	32,7	3,9	8,6
2021q1	34,2	16,1	30,8	3,4	13,7	2,6	9,6	31,7	33,8	32,2	34,2	34,1	32	31,2	31,7	32,8	4	8,7
2021q2	34,2	16,1	30,8	3,4	13,8	2,6	9,6	31,8	33,9	32,2	34,3	34,2	32,1	31,4	31,7	32,9	4	8,7
2021q3	34,3	16,1	30,4	3,4	13,9	2,6	9,6	32	33,9	32,2	34,4	34,2	32,2	31,4	31,9	32,9	3,8	8,7
2021q4	34,3	16,2	31,1	3,4	13,9	2,6	9,6	32,1	34	32,4	34,5	34,3	32,3	31,5	31,9	33,1	4	8,8
2022q1	34,3	16,2	30,9	3,4	13,9	2,6	9,6	31,8	34,1	32,4	34,5	34,3	32,3	31,5	32	33,1	4	8,8
2022q2	34,3	16,2	31,3	3,4	14	2,6	9,6	31,9	34,1	32,4	34,6	34,4	32,3	31,8	32,1	33,1	3,9	8,9

## Appendix 1

CLIs of Data Transformation.

## Appendix 2

CLIs of Data Transformation and Seasonal Adjustment.

## Appendix 3

**Paramater Measured Comparison**.

**R2 and R2 Ajusted Model Comparison**.

**Model Fit Summary Comparison**.

## Appendix 4

**IEWS Structural Model 1 & 5 vs CLIs t-k**.

## Appendix 5

**IEWS PLS – SEM Individual CLI vs Linear Model**.

## Appendix 6

**IEWS PLS – SEM Individual CLI t-k vs Linear Model**.
